# Five-year Prognosis after Mild to Moderate Ischemic Stroke by Stroke Subtype: A Multi-Clinic Registry Study

**DOI:** 10.1371/journal.pone.0075019

**Published:** 2013-11-04

**Authors:** Yumei Lv, Xianghua Fang, Karam Asmaro, Hongjun Liu, Xinqing Zhang, Hongmei Zhang, Xiaoming Qin, Xunming Ji

**Affiliations:** 1 Evidence-based Medical Center, Xuanwu Hospital, Capital Medical University, Beijing, China; 2 School of Nursing, Harbin Medical University (Daqing), Daqing, Heilongjiang Province, China; 3 Department of Neurological Surgery, Wayne State University School of Medicine, Detroit, Michigan, United States of America; 4 Department of Neurology, Xuanwu Hospital, Capital Medical University, Beijing, China; 5 Department of Geriatrics, Xuanwu Hospital, Capital Medical University, Beijing, China; 6 Department of Neurological Surgery, Xuanwu Hospital, Capital Medical University, Beijing, China; University Medical Center Utrecht, The Netherlands

## Abstract

**Background and Purpose:**

Mild to moderate ischemic stroke is a common presentation in the outpatient setting. Among the various subtypes of stroke, lacunar infarction (LI) is generally very common. Currently, little is known about the long-term prognosis and factors associated with the prognosis between LI and non-LI. This study aims to compare the risk of death and acute cardiovascular events between patients with LI and non-LI, and identify potential risk factors associated with these outcomes.

**Methods:**

A total of 710 first-ever ischemic stroke patients (LI: 474, non-LI: 263) from 18 clinics were recruited consecutively from 2003 to 2004. They were prospectively followed-up until the end of 2008. Hazard ratios and 95% confidence intervals were calculated using multivariable Cox proportional hazards regression.

**Results:**

After a 5-year follow up, 54 deaths and 96 acute cardiovascular events occurred. Recurrent stroke was the most common cause of death (19 cases, 35.18%) and new acute cardiovascular events (75 cases, 78.13%). There were no significant differences between patients with LI and non-LI in their risks of death, new cardiovascular events, and recurrent stroke after adjusting for age, sex, hypertension, diabetes, cardiac diseases, body mass index, dyslipidemia, smoking, alcohol consumption, ADL dependence, and depressive symptoms. Among the modifiable risk factors, diabetes, hypertension, ADL dependency, and symptoms of depression were independent predictors of poor outcomes in patients with LI. In non-LI patients, however, no modifiable risk factors were detected for poor outcomes.

**Conclusion:**

Long-term outcomes did not differ significantly between LI and non-LI patients. Detecting and managing vascular risk factors and depression as well as functional rehabilitation may improve the prognoses of LI patients.

## Introduction

Stroke is one of the leading causes of death and disability worldwide. Recent epidemiological studies have shown a significant increase in the number of stroke survivors in China [1]. Along with the burden of cardiovascular disease, stroke survivors are at high risks of recurrence and ischemic heart disease. The neurological sequelae that follow a recurrent stroke event can be debilitating, and recurrent stroke patients carry greater risks of disability and death [2], [3], [4]. Secondary prevention of stroke continues to be a challenge and is of great concern to clinicians. Studies have shown that comprehensive modification of the stroke-related risk factors in combination with antiplatelet therapy may prevent recurrent stroke [5]. To identify reliable long-term prognostic indicators in ischemic stroke patients could allow clinicians to find patients with high risk for non-fatal or fatal vascular events. More importantly, a thorough understanding of prognostic indicators would help investigators design more effective intervention measures for prophylaxis against primary or recurrent stroke events.

In China, several studies have attempted to evaluate the prognosis of ischemic stroke and risk factors associated with poor outcomes [6], [7], [8]. The majority of the studies, however, focused on hospitalized patients or inpatients in the acute stage of stroke and the duration of follow-up was less than 18 months. Currently, there is paucity in the literature regarding the outcome of patients who experience a mild to moderate stroke in the post-acute stage after being treated in the outpatient setting or discharged from the hospital. This group of patients usually prefers to see a physician in clinics located in their community for post-stroke treatment and rehabilitation. An earlier study showed that the most common subtype of stroke diagnosed in patients who present to clinics differed from that diagnosed inpatients who were already hospitalized [6], [7], [8]. In the outpatient settings, patients most often present with lacunar infarction (LI) [9]. Finally, long-term prognosis, as well as factors which might influence prognosis in patient with LI and non-LI are not clearly described.

In present study, we examined the 5-year risks of death, acute cardiovascular events as well as recurrent stroke in Chines patients with mild to moderate ischemic stroke in primary clinics. The majority patients had a subtype stroke of Lacunar infarction. We aimed to 1) compare the observed risks of death, acute cardiovascular events as well as recurrent stroke in LI patients to those in non-LI patients; 2) to identify potential risk factors which predict death, acute cardiovascular and recurrent stroke in both LI and non-LI patients.

## Methods

### Patient Selection and Study Design

In China, hospitals are classified into three grades; primary clinics (community hospitals) are defined as grade I, hospitals that serve several communities are defined as grade II, and central hospitals for a certain district or city are defined as grade III and are usually teaching hospitals [10]. There are 600 primary clinics in the Beijing municipality. We performed a prospective registry study in 18 primary clinics which were selected randomly from locations in Eastern, Southern, Western, Northwestern, and Northeastern regions of urban Beijing, China. The study was were approved by the ethics committee of Xuanwu Hospital, Capital Medical University and written informed consent was obtained from the study subjects. A total of 1,371 stroke patients were recruited consecutively between December 2003 and December 2004. Patients eligible for enrollment were those who presented with a symptomatic stroke event, were diagnosed by a neurologist, and underwent examinations of computed tomography (CT) or magnetic resonance imaging (MRI). Patients with any silent stroke or who experienced an acute stroke within 3 months were excluded from the study. Of the 1,371 patients, 1,308 had experienced an ischemic stroke and 63 hemorrhagic stroke. Of the 1,308 ischemic stroke patients, a total of 134 of patients did not meet enrollment criteria, and 86 cases refused to take part in the study as well as to provide written consents. Therefore, 1,088 consented to participate in the study and were enrolled, 710 cases presented with a first-ever ischemic stroke. Here we reported these 710 patients.

All enrolled patients underwent a standardized assessment by a multidisciplinary stroke care research team, including physicians, neurologists, epidemiologists, and nurses. To ensure uniformity of research methods, a manual of operations was compiled to standardize the research methods and procedures across the clinics. All of the people participated in the study were trained before the start of the study (See File S1). The assessment included gathering information on demographic characteristics, all known risk factors for cerebrovascular diseases, including hypertension, diabetes, and dyslipidemia. Smoking and drinking practices were characterized as never, current, or quit. Disability was assessed with the Barthel Index (BI) [11]. The BI evaluates 10 basic activities of self-care (feeding, grooming, dressing, toileting, bathing, bowel and bladder continence) and mobility (transferring, walking, and stair climbing) on a total functional score from 0 (totally dependent) to 100 (totally independent). Depressive symptoms were assessed using the Self-Rating Depression Scale (SDS). The SDS is a 20-item, self-reported index of the frequency of experienced depressive symptoms. This scale has been shown to have a sensitivity of 97% and specificity of 63% for depressive disorders in a general medical clinic according to the *Diagnostic and Statistical Manual of Mental Disorders*, 3^rd^ edition [12]. Cutoff scores for the SDS were: <50 (normal), 50 to 59 (mild), 60 to 69 (moderate), and >69 (severe). Depression scales were administered by research physicians trained by a psychiatrist specialized in the assessment and management of post-stroke mood disorders.

After the baseline assessment, the patients were interviewed every six months until the end of December 2008. At each follow-up interview, physicians in clinics spoke directly with the patients or their family members in order to document any acute events pertaining to cardiovascular events defined as stroke recurrence, acute myocardial infarction (AMI), and sudden death occurring in the previous six months. Hospital medical records were obtained in order to confirm the reported events. By the end of December 2008, 639 patients were successfully followed-up. The drop-out rate was 10% (71/710), mostly due to patients' relocation.

Diagnosis of recurrent stroke was made by neurologists and were based on clinical symptoms, physical signs, and CT/MRI findings. The classifications of LI and non-LI were based on the diagnosis at discharge from a hospital or the outpatient-clinic medical record.

The risk factors were defined as follows: hypertension (reported systolic blood pressure [SBP] ≥140 mmHg, reported diastolic blood pressure [DBP] ≥90 mmHg, self-reported hypertension by the patient, or use of antihypertensive drugs), cardiac disease (history of myocardial infarction, coronary artery disease, congestive heart failure, arrhythmia, or valvular heart disease), diabetes mellitus (fasting blood glucose level ≥7.0 mmol/L, self-reported diabetes by the patient, or use of anti-diabetic drugs), dyslipidemia (fasting total cholesterol ≥5.72 mmol/L, low-density lipoprotein ≥3.64 mmol/L, high-density lipoprotein ≥0.91 mmol/L, triglyceride ≥1.70 mmol/L, self-reported history of hyperlipidemia by the patient, or use of antihyperlipidemic drugs). Body mass index (BMI) was calculated according to the formula: body weight (kg)/[body height (m)]^2^.

### Statistics

Age was treated as a continuous variable. Sex, vascular risk factors, and study end points were treated as binary variables. We also treated BI (<95 vs.≥95) and SDS scores as categorical variables. Differences between LI and non-LI were assessed with Student's *t* test for continuous variables and the 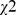 test for proportions.

In survival analyses, the Kaplan-Meier product-limit method was used to estimate survival rate. The log-rank test was used to compare rate estimates. The Cox proportional hazards regression function was used to estimate impact in terms of risk ratios of possible determinants for death and acute CVD, respectively. The hazard ratio (HR), 95% confidence interval (CI), and probability value were calculated.

## Results

Among the 710 cases of first ever ischemic stroke, 474 (66.8%) experienced a LI. The average age of those patients was 64.4±9.3 years. The prevalence of hypertension, diabetes, dyslipidemia, and cardiac disease in those patients were 77.6%, 30.1%, 72.1%, and 34.4%, respectively (Table 1). The average age, SBP, BI score, male gender, prevalence of hypertension and depression, as well as the percentage of male patients and the percentage of patients independent in their ADLs were significantly lower in the LI group than in the non-LI group (P<0.05). The percentage of patients being prophylactically treated with aspirin was significantly lower in the LI group than non-LI group (69.0% vs. 76.3%, P = 0.043).

**Table 1:** Baseline characteristics in LI and non-LI patients 3-month after first ischemic stroke.

Variables	Overall	LI	Non-LI		P-value	t/ 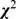
	(N = 710)	(N = 474)	(N = 236)			
Age±SD (year)	64.4±9.3	63.7±8.9	65.9±9.7		0.003	3.031
Male,% (n)	51.0 (362)	46.4 (220)	60.2 (42)		<0.0001	11.930
SBP±SD (mmHg)	135.1±16.0	133.9±15.3	137.6±17.2		0.003	2.955
DBP±SD (mmHg)	81.7±9.8	81.2±9.7	82.7±10.0		0.057	1.903
FBG±SD (mmol/L)	6.0±2.0	6.063±2.100	5.959±1.651		0.515	0.939
TC±SD (mmol/L)	5.181±1.060	5.205±1.043	5.133±1.089		0.398	0.845
TG±SD (mmol/L)	1.835±1.376	1.809±1.490	1.895±0.112		0.496	0.681
HDL-C±SD (mmol/L)	1.254±0.382	1.256±0.391	1.249±0.362		0.827	0.219
LDL-C±SD (mmol/L)	3.106±0.997	3.145±0.961	3.027±1.063		0.143	1.466
Hypertension,% (n)	77.6(551)	74.5 (353)	83.9 (198)		0.005	8.054
Diabetes,% (n)	30.1 (208)	28.9 (137)	30.1 (71)		0.744	0.106
Dislipidemia (%)	72.1 (512)	71.1 (337)	74.2 (175)		0.424	0.731
BMI±SD (kg/m^2^)	25.4±3.2	25.3±3.5	25.6±3.5		0.332	0.971
Cardiac diseases,% (n)	34.4 (244)	36.1 (171)	30.9 (73)		0.174	1.848
*Smoking*,% (n) Never	65.6 (466)	67.5 (320)	61.9 (146)		0.095	4.700
Current	14.8 (105)	15.2 (72)	14.0 (33)			
Quit	19.6 (139)	17.3 (82)	24.2(57)			
*Alcohol drinking*,% (n) Never	71.8 (510)	71.9 (341)	71.6 (169)		0.049	6.047
Current	14.8 (105)	16.5 (78)	11.4 (27)			
Quit	13.4 (93)	11.6 (55)	16.9 (38)			
BI index (Average score)	94.2±14.7	96.6±10.5	89.3±19.9		<0.0001	6.365
ADL totally independence,% (n)	28.3 (201)	20.5 (97)	44.1 (104)		<0.0001	43.251
Depressive symptoms,% (n)	45.3 (322)	40.5 (192)	55.1 (130)		<0.0001	14.840
Severity of depressive symptoms,% (n)						
No	21.4 (150)	20.2 (95)		23.8 (55)	0.002	14.549
Mild	16.4 (115)	13.8 (65)		21.6 (50)		
Moderate or severe	4.3 (30)	3.4 (16)		6.1 (14)		
Aspirin treatment,% (n)	71.4 (507)	69.0 (327)		76.3 (180)	0.043	4.094

IS, Ischemic stroke; LI, Lacunar Infarction; non-LI, non-lacunar infarction, including thrombotic brain infarction and cardioembolic stroke; SBP, Systolic blood pressure; DBP, diastolic blood pressure; FBG, fasting blood glucose; TC, Total cholesterol; TG, Triglyceride; HDL-C, High-density lipoprotein cholesterol; LDL-C, Low-density lipoprotein cholesterol; BMI, Body mass index; BI, Bathel index.

**Figure 1:**
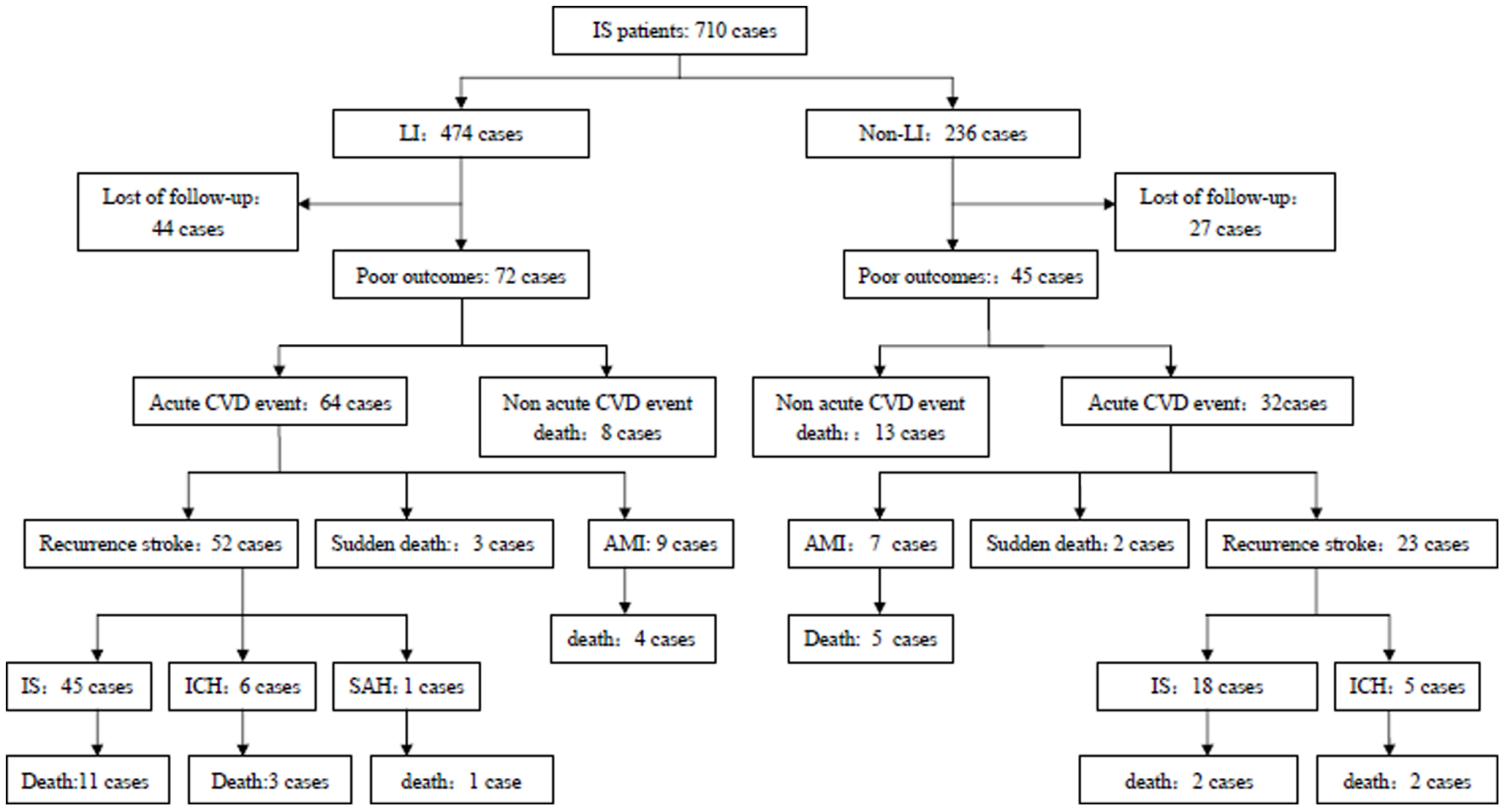
Prognosis of 710 patients with first ischemic stroke. LI, Lacunar Infarction; non-LI, non-lacunar infarction, including thrombotic brain infarction and cardioembolic stroke; Acute CVD, acute cardiovascular diseases including acute myocardial infarction, sudden death and acute stroke; AMI, acute myocardial infarction; IS, Ischemic stroke; ICH, intracerebral hemorrhage; SAH, subarachnoid hemorrhage.

By the end of 2008, a total of 2,477 person-years were followed (LI: 1699 person-years; non-LI: 778 person-years). The mean duration of follow-up was 3.5±0.9 person-years (range: 0.5 to 5 years). Fifty-four deaths and 96 new onset acute cardiovascular events occurred (Figure 1). Recurrent stroke was the most common cause of death (19 cases, 35.18%). In addition, 9 deaths were caused by AMI, and 5 by sudden death. The remaining 21 patients died of non-acute cardiovascular events, including pulmonary infection (7 deaths), tumor (6 deaths), renal failure (2 deaths), gastrointestinal bleeding (1 death), and other causes (5 deaths). Recurrent stroke was also the major causes of acute cardiovascular events. Among the 96 new acute cardiovascular events, 75 (78.3%) had recurrent stroke; 16 were AMI and 5 sudden deaths.

Among those patients who died, 30 were in the LI group, while. 24 were in the non-LI group. Similarly, among those who experienced an acute cardiovascular event, 64 were in the LI group, while only 32 were in the non-LI group (Figure 1). Compared with the non-LI group, the proportion of recurrent stroke was higher in the LI group (81.3% in LI vs. 71.9% in non-LI). Acute cardiovascular events, including hemorrhagic stroke, AMI, and sudden death, which were generally more likely to be fatal or have severe clinical outcomes, were more common in patients with non-LI than LI. However all of these differences were not statistically significant.

**Figure 2:**
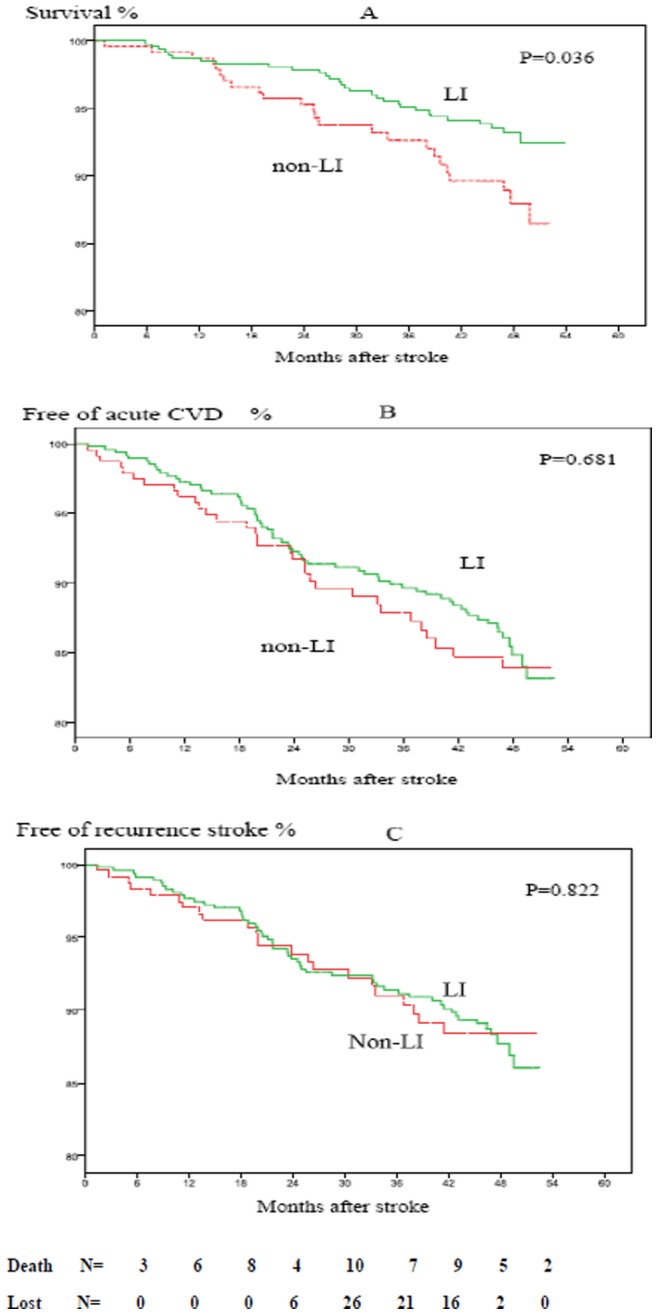
Kaplan-Meier estimates on observed percentages of surviving (A), free of acute CVD (B) and free of recurrent stroke (C) after incidence of first ischemic stroke in patients with LI and non-LI. LI, Lacunar Infarction; non-LI, non-lacunar infarction, including thrombotic brain infarction and cardioembolic stroke; Acute CVD, acute cardiovascular diseases including acute myocardial infarction, sudden death and acute stroke; AMI, acute myocardial infarction; IS, Ischemic stroke; ICH, intracerebral hemorrhage; SAH, subarachnoid hemorrhage.

Survival curves of cumulative risk of death, acute cardiovascular events, and recurrent stroke in patients with LI and non-LI are presented in Figure 2. Although cumulative mortality in LI patients was significantly lower than that in non-LI patients (8.8% vs. 14.8%, *P* = 0.035), (Table 2), a significant difference was no longer presented after adjusted for baseline age, sex, hypertension, diabetes, cardiac diseases, BMI, hyperlipidemia, smoking, alcohol consumption, depressive symptoms, and ADL status. Similar results were found in univariate and multivariate analysis by COX regression models when cardiovascular events or recurrent stroke were used as endpoints.

**Table 2:** 5-year cumulative mortality rate, incident rates of acute CVD and recurrent stroke, HR and 95% CI (non-LI vs LI).

Endpoints	No of Death/event	5-yr Cum rate	Crude HR		Adjusted HR*	
			HR and 95% CI	Wald/P-value	HR and 95% CI	Wald/P-value
All-cause death						
Non-LI	24	10.2	1		1	
LI	30	6.3	0.56 (0.33–0.96)	4.28/0.035	1.17 (0.61–2.25)	0.213/0.642
Acute CVD event						
Non-LI	32	13.6	1		1	
LI	64	13.5	0.91 (0.59–1.38)	0.168/0.644	1.27 (0.78–2.07)	0.532/0.466
Recurrent stroke						
Non-LI	23	9.7	1		1	
LI	53	11.2	1.05 (0.64–1.71)	0.051/0.853	1.43 (0.82–2.48)	1.368/0.204

* Adjusted for age, gender, hypertension, diabetes, dislipidemia, cardiac disease, overweight or obesity, smoking status, drinking status, BI index, depression symptoms, aspirin treatment. LI, Lacunar Infarction; non-LI, non-lacunar infarction, including thrombotic brain infarction and cardioembolic stroke; Acute CVD, acute cardiovascular diseases including acute myocardial infarction, sudden death and recurrent stroke; Recurrent stroke including ischemic stroke, intracerebral hemorrhage and subarachnoid hemorrhage; HR, Hazard ratio; CI, Confidence interval.

Among the 474 LI patients (Table 3), the risks of death, acute cardiovascular events and recurrent stroke were 70% (HR = 0.3, 95% CI: 0.10–0.88, P = 0.029),52% (HR = 0.48, 95% CI: 0.26–0.91, P = 0.025), and 54% (HR = 0.46, 95% CI: 0.22–0.95, P = 0.037) lower in female than in male, respectively. The risks of acute cardiovascular events and recurrent stroke were 1.78 times (HR:1.78, 95% CI: 1.01–3.15, P = 0.047) and 1.98 times (HR:1.98, 95% CI: 1.18–3.31, P = 0.010) higher, respectively, in diabetics when compared to non-diabetics. ADL dependence was also independently associated with the risks of death (HR: 6.67, 95% CI: 2.63–10.87, P<0.0001) and acute cardiovascular events (HR: 1.97, 95% CI: 1.10–3.53, P = 0.022). In addition, age (HR: 1.09, 95% CI: 1.02–1.16, P = 0.007), hypertension (HR: 1.35, 95% CI: 1.14–1.19, P = 0.031), and moderate to severe depression symptom (HR: 3.25, 95% CI: 1.28–8.27, P = 0.013). were also independent predictors of the risk of death. In comparison to patients with normal mood, the risk of recurrent stroke increased by 1.96 times (HR: 1.96, 95% CI: 1.02–3.77, P = 0.043) in the patients with mild depression; and the risk of death increased by 3.3 times (HR: 3.25, 95% CI: 1.28–8.27, P = 0.013) in the patients with moderate to severe depression. In non-LI patients, however, no modifiable risk factors were associated with poor outcomes (see Table S1).

**Table 3:** Survival models for determinants of 5-year mortality, incidence of acute CVD events and recurrent stroke events in LI.

	All-cause death		Acute CVD event		Recurrent stroke	
Factors 3-month after first ever stroke	Adjusted HR and 95% CI*	Wald/P value	Adjusted HR and 95% CI *	Wald/P value	Adjusted HR and 95% CI*	Wald/P value
Age (per year)	1.09 (1.02–1.16)	8.334/0.007	1.03 (1.00–1.07)	3.252/0.067	1.02 (0.99–1.06)	1.373/0.250
Gender (male vs female)	0.30 (0.10–0.88)	5.632/0.029	0.48 (0.26–0.91)	5.068/0.025	0.46 (0.22–0.95)	4.370/0.037
Hypertension (no vs yes)	1.35 (1.14–1.91)	4.943/0.031	1.33 (0.62–2.83)	0.477/0.461	1.48 (0.63–3.46)	0.796/0.371
Diabetes (no vs yes)	1.70 (0.73–3.96)	0.313/0.221	1.98 (1.18–3.31)	6.632/0.010	1.78 (1.01–3.15)	4.069/0.047
Dislipidemia (no vs yes)	0.50 (0.22–1.12)	2.220/0.091	0.72 (0.42–1.23)	1.439/0.231	1.02 (0.54–1.91)	0.001/0.960
Cardiac diseases (no vs yes)	2.96 (1.21–7.22)	5.654/0.017	1.47 (0.86–2.51)	1.966/0.160	1.37 (0.75–2.47)	1.080/0.304
Overweight or obese (no vs yes)	1.31 (0.55–3.13)	0.192/0.539	1.24 (0.70–2.20)	0.480/0.460	1.11 (0.59–2.08)	0.117/0.742
Smoking Never	1		1		1	
Current	1.78 (0.49–6.46)	0.486/0.381	1.19 (0.52–2.70)	0.098/0.679	0.84 (0.33–2.12)	0.130/0.717
Quit	1.79 (0.60–5.33)	1.004/0.299	1.01 (0.46–2.18)	0.007/0.988	0.82 (0.35–1.93)	0.241/0.643
Alcohol drinking Never	1		1		1	
Current	1.11 (0.31–3.96)	0.026/0.873	0.70 (0.29–1.70)	0.477/0.428	1.07 (0.42–2.71)	0.023/0.889
Quit	0.79 (0.24–2.59)	0.079/0.693	1.06 (0.46–2.42)	0.048/0.899	1.66 (0.69–4.02)	1.245/0.260
BI index (totally independence vs dependence)	6.67 (2.63–10.87)	20.274/<0.0001	1.97 (1.10–3.53)	5.788/0.022	1.87 (0.98–3.57)	3.531/0.057
Severity of depressive symptoms						
No	1		1		1	
Mild	0.64 (0.19–2.18)	0.447/0.476	1.48 (0.80–2.75)	1.593/0.212	1.96 (1.02–3.77)	4.077/0.043
Moderate or severe	3.25 (1.28–8.27)	5.642/0.013	1.47 (0.76–2.87)	1.265/0.255	1.30 (0.59–2.86)	0.378/0.521

* Adjusted for age, gender, hypertension, diabetes, dislipidemia, cardiac disease, overweight or obesity, smoking status, drinking status, BI index, depression symptoms LI, Lacunar Infarction; Acute CVD, acute cardiovascular diseases including acute myocardial infarction, sudden death and acute stroke; Recurrent stroke including ischemic stroke, intracerebral hemorrhage and subarachnoid hemorrhage. HR, Hazard ratio; CI, Confidence interval; BI, Bathel index.

## Discussion

In our study, no significant differences in the 5-year cumulative risks of acute cardiovascular events (including stroke recurrence) were noted between first-ever LI and non-LI patients. After adjustment for baseline confounders, the risk of death for patients with LI and non-LI was similar to non-LI. Previously, in a community-based study, we found that the major difference in mortality between LI and non-LI patients was in the acute stage of stroke. The disparity in the mortality rates of the two groups decreased from one to 12 months of acute stroke onset, and disappeared after one year [13]. Due to limitaltions in its initial design, the aforementioned study was unable to identify long-term risks for vascular endpoints and to analyze potential risk factors associated with death and acute cardiovascular events. The benefit of identifying the various possible outcomes and risk factors in post-ischemic stroke survivors allows the medical and scientific community to develop strategies aiming at reducing the burden of stroke on patients and their families, and by doing so increase quality as well as quantity of life in these patients. The results from this study reaffirm the notion that the post-stroke risks of acute cardiovascular events and mortality are higher than those of the general population [3]. A recent report from urban Beijing showed that the average incidence of first stroke in general population was 0.178% with 95% CI of 0.113% to 0.131% during the period of 2003 to 2008 [13].

Our study demonstrated stroke recurrence to be the most common cause of death, followed by AMI in ischemic stroke survivors. In contrast, a meta-analysis by Sander *et al*. found that stroke survivors to be at great risk of recurrent stroke in the short term, while AMI posed a greater risk in the long term [14]. However, most studies in Sander and colleagues' report encompassed a European or American patient populations. Japanese scholars speculated that the risk factors for recurrent stroke in East Asian populations were different from those of European or American stroke survivors [15]. Heterogeneity from a genetic or environmental standpoint and the interaction between the two might explain the reason for the disparity between the different populations. Another explanation might be that intracranial large-artery occlusive disease, the most common cause of stroke, is more prevalent in the Chinese population when compared to the Caucasian one [16].

The risk factor profiles associated with poor outcome were different between patients with LI and non-LI. Several significant predictors of poor outcome in LI patients were identified. They were age, male gender, diabetes, hypertension, ADL dependence, and depression. These findings are consistent with previous studies from China [6], [7], [8], [9], [17]. The majority risk factors we identified to be prognostic of poor outcomes in LI patients are modifiable (only age and gender are fixed). These findings suggested that as long as the vascular risk factors controlling, as well as rehabilitation and psychological counseling are aggressively administrated, the prognosis of LI patients can possibly be shifted in a favorable direction. A meta-analysis by Hankam *et al*. showed that the risk of recurrent stroke could be reduced by 80% when stroke survivors were actively treated for carotid artery atherosclerosis, atrial fibrillation, diabetes, and combined anticoagulant therapy and smoking cessation [5]. Improving functional ability and depression symptoms are also of benefit to stroke survivors [18], [19]. In China, current guidelines for the secondary prevention of stroke put emphased of management of vascular risk factors; unfortunately, though, the rehabilitation and mental health care are not adequately stressed [20].

To our knowledge, this present study is the first large sample, long-term study focuses on multiple end-points and determinants related to the poor outcomes of stroke in mild to moderate ischemic stroke survivors in clinics in China. Due to limited resources in developing countries, the admission to a hospital after a stroke event depends mainly on the severity of the disease. Patient with a severe presentation is more likely to be admitted [21]. As a result, hospital-based studies from developing countries are usually biased towards the more serious or complicated cases. In addition, a recent study showed that, in China, a patient's socioeconomic status also influences health providers' decisions regarding admission and treatment [22]. Patients with a mild to moderate stroke or those whose healthcare are not covered by medical insurance would often present to the outpatient clinics for treatment. The baseline assessment in our study suggested that the demographic characteristics, vascular risk profiles, and the proportion of ischemic stroke subtype in clinics were different from those of inpatient services or who are in the acute stage of stoke. Compared with previous hospital-based studies, [6], [7], [8], the proportion of female gender and the prevalences of hypertension and dislipidemia were higher in our clinics' patients. The proportion of LI was 66.8% (474/710), while the proportion was 42.3% in hospital-based study cover 5 hospitals in China [7] Because the patients who present to the clinics are generally stable, their prognosis have been paid less attention in China now. According to our study, the strategies that are utilized for the inpatients in the acute stage of stroke should be adjusted if those are to be use in the outpatient in the stable phase of the disease in clinics. Hence, a well-organized and professional drive is needed for vascular risk management and rehabilitation. The results from this study might be of importance to clinical and public policy changes related to stroke prevention, rehabilitation, and long-term care.

Worth mentioning are several limitations presented in this study. It is possible that the cohorts we selected for the study were not be true representatives of the mild to moderate ischemic stroke patient subpopulation that presents to clinics for treatment in China. To ensure the accuracy of our diagnosis and classification of ischemic stroke, we excluded those patients whose diagnosis of stroke was not confirmed by neuroimaging. Secondly, we did not find significant difference in the proportion of hemorrhagic stroke, AMI, and sudden death between LI and non-LI groups. In addition, we also did not identify any modifiable risk factors related to poor prognoses in non-LI patients. These negative findings could be attributed to a relatively small number of non-LI patients enrolled at baseline and thereafter a small number of new-onset acute cardiovascular events occurring in this group during the follow-up period; thereby decreasing the power for detecting statistical significance. Clearly, in order to properly assess outcomes and identify underlying determinants of prognoses, a large long-term community-based study with adequate representation of the different subtypes of stroke is needed. Thirdly, the dropout rate among this study's participants was 10%, mainly due to participants' relocation. Fortunately, the baseline characteristics between patients lost to follow-up and those remained in the cohort were similar (data not shown), and dropout rates between LI and non-LI were similar (9.3% vs.11.4%, P = 0.219), which suggests that the impact of patients lost to follow-up is limited. Fourthly, at the baseline data collection, we only asked whether of not patients used tobacco or alcohol prior to their index stroke. Quantitative data (amount and duration of use) were not collected. Therefore the association between the use of tobacco or alcohol and poor outcomes may be underestimated, as patients experiencing a stroke are more likely to give up such habits.

## Supporting Information

File S1 **The contents of raining for the doctors and nurses who participated the study.**
(DOCX)

Table S1 **Survival models for determinants of 5-year mortality, incidence of acute CVD events and recurrent stroke events in non-LI.**
(DOCX)
