# Supplementary material for: Five-year Prognosis after Mild to Moderate Ischemic Stroke by Stroke Subtype: A Multi-Clinic Registry Study
Source: PLoS One. 2013 Nov 4;8(11):e75019. doi: 10.1371/journal.pone.0075019 (PMC3817184; doi:10.1371/journal.pone.0075019)
Supplement: File S1 — The contents of raining for the doctors and nurses who participated the study. (DOCX) [file pone.0075019.s001.docx]

A steering committee composed of the principal investigators was responsible for the scientific leadership of the study. To ensure uniformity of research methods, a manual of operations was compiled to standardize the research methods and procedures across the clinics. The contents included the criteria for patient enrollment, how to interview the subjects and perform physical examinations, follow up subjects for the risk factors of stroke, report and monitor new cases of stroke, and the criteria for the diagnosis of stroke. All workers who participated in the study were required to receive training in the basics of the study’s design and conduct and to be familiar with the manual of operations before participating in the study. An annual meeting was held with GP from the primary clinics to review the research progress and address problems encountered
