# Supplementary material for: Five-year Prognosis after Mild to Moderate Ischemic Stroke by Stroke Subtype: A Multi-Clinic Registry Study
Source: PLoS One. 2013 Nov 4;8(11):e75019. doi: 10.1371/journal.pone.0075019 (PMC3817184; doi:10.1371/journal.pone.0075019)
Supplement: Table S1 — Survival models for determinants of 5-year mortality, incidence of acute CVD events and recurrent stroke events in non-LI. (DOCX) [file pone.0075019.s002.docx]

Table S. Survival models for determinants of 5-year mortality, incidence of acute CVD events and recurrent stroke events in non-LI

| Factors 3-month | All-cause death | |  | Acute CVD event | |  | Recurrent stroke | |
| --- | --- | --- | --- | --- | --- | --- | --- | --- |
| after first ever stroke | Adjusted HR and 95% CI* | wald/P value |  | Adjusted HR and 95% CI * | wald/P value |  | Adjusted HR and 95% CI* | wald/P value |
| Age(per year) | 1.17(1.08-1.27) | 15.706/<0.0001 |  | 1.02(0.98-1.07) | 1.118/0.378 |  | 0.99(0.95-1.05) | 0.000/0.924 |
| Gender(male vs female) | 1.07(0.30-3.75) | 0.067/0.920 |  | 1.24(0.46-3.37) | 0.055/0.670 |  | 1.07(0.34-3.40) | 0.052/0.906 |
| Hypertension(no vs yes) | 1.39(0.28-6.80) | 0.152/0.684 |  | 3.24(0.73-14.35) | 2.541/0.122 |  | 4.97(0.63-38.97) | 2.527/0.127 |
| Diabetes(no vs yes) | 1.97(0.75-5.21) | 1.653/0.170 |  | 1.99(0.95-4.18) | 3.838/0.069 |  | 2.56(1.06-6.15) | 5.232/0.036 |
| dislipidemia(no vs yes) | 1.04(0.37-2.92) | 0.003/0.938 |  | 1.44(0.56-3.68) | 0.892/0.444 |  | 1.36(0.43-4.34) | 0.613/0.601 |
| Cardiac diseases (no vs yes) | 0.75(0.29-1.99) | 0.250/0.569 |  | 1.22(0.56-2.64) | 0.015/0.614 |  | 0.91(0.35-2.39) | 0.148/0.852 |
| Overweight or obese(no vs yes) | 0.45(0.18-1.12) | 2.747/0.086 |  | 0.90(0.39-2.05) | 0.407/0.796 |  | 1.22(0.42-3.55) | 0.015/0.717 |
| Smoking Never | 1 |  |  | 1 |  |  | 1 |  |
| Current | 0.43(0.05-3.82) | 0.538/0.453 |  | 0.41(0.08-2.05) | 1.353/0.276 |  | 0.48(0.09-2.67) | 0.806/0.401 |
| Quit | 1.27(0.39-4.09) | 0.149/0.692 |  | 1.29(0.47-3.55) | 0.046/0.624 |  | 1.04(0.30-3.57) | 0.010/0.948 |
| Alcohol drinking Never | 1 |  |  | 1 |  |  | 1 |  |
| Current | 2.4(0.57-10.15) | 1.702/0.234 |  | 1 | 1.997/0.115 |  | 2.02(0.51-7.98) | 0.611/0.315 |
| Quit | 2.41(0.60-9.76) | 2.050/0.217 |  | 1.74(0.50-6.01) | 0.646/0.381 |  | 1.48(0.35-6.36) | 0.098/0.595 |
| BI index（totally independence  vs dependence） | 1.52(0.55-4.18) | 0.620/0.416 |  | 0.82(0.36-1.84) | 0.005/0.629 |  | 0.96(0.37-2.48) | 0.015/0.929 |
| Severity of depressive symptoms |  |  |  |  |  |  |  |  |
| No | 1 |  |  | 1 |  |  | 1 |  |
| Mild | 1.25(0.35-4.44) | 0.065/0.727 |  | 1.38(0.57-3.34) | 1.521/0.471 |  | 1.87(0.68-5.14) | 2.462/0.224 |
| Moderate or severe | 0.69(0.20-2.34) | 0.347/0.552 |  | 0.59(0.21-1.67) | 0.859/0.324 |  | 0.73(0.21-2.56) | 0.262/0.623 |

*Adjusted for age, gender, hypertension, diabetes, dislipidemia, cardiac disease, overweight or obesity, smoking status, drinking status, BI index,

depression symptoms

LI, Lacunar Infarction; Acute CVD, acute cardiovascular diseases including acute myocardial infarction, sudden death and acute stroke; Recurrent stroke including ischemic stroke, intracerebral hemorrhage and subarachnoid hemorrhage. HR, Hazard ratio; CI, Confidence interval; BI, Barthel index.
